# Dapagliflozin Attenuates Myocardial Fibrosis by Inhibiting the TGF-β1/Smad Signaling Pathway in a Normoglycemic Rabbit Model of Chronic Heart Failure

**DOI:** 10.3389/fphar.2022.873108

**Published:** 2022-05-13

**Authors:** Xuefeng Chen, Qian Yang, Wenlou Bai, Wenjing Yao, Litian Liu, Yuanyuan Xing, Cunliang Meng, Peng Qi, Yi Dang, Xiaoyong Qi

**Affiliations:** ^1^ Department of Internal Medicine, Hebei Medical University, Shijiazhuang, China; ^2^ Department of Cardiology Center, Hebei General Hospital, Shijiazhuang, China

**Keywords:** SGLT2 inhibition, dapagliflozin, myocardial fibrosis, chronic heart failure, rabbit model, TGFβ1/Smad

## Abstract

Recent studies have shown that sodium-glucose cotransporter-2 (SGLT2) inhibitors play a beneficial role for normoglycemic patients with heart failure (HF). However, the underlying mechanism remains largely unexplored. In the present study, we aimed to investigate the cardioprotective effect of SGLT2 inhibitors in a normoglycemic rabbit model of chronic heart failure (CHF) and its potential mechanism was also explored. A total of 24 male New Zealand white rabbits were randomly divided into the sham group, HF group, perindopril group, and dapagliflozin (DAPA) group. The normoglycemic CHF model was established by aortic constriction for 12 weeks. In the 13th week, DAPA (1 mg/kg/day) or perindopril (0.5 mg/kg/day) was administered by oral gavage daily for 10 weeks. Both the sham group and HF group were given normal saline *via* gavage. After 10 weeks, the heart structure and function were evaluated by echocardiography and plasma NT-proBNP. Moreover, cardiac fibrosis was analyzed using immunohistochemistry, Masson’s trichrome staining, and Western blotting analysis. The results showed that DAPA improved the myocardial structure and function of normoglycemic CHF rabbits and ameliorated myocardial fibrosis. Further study indicated that DAPA suppressed cardiac fibrosis by inhibiting the transforming growth factor β1 (TGF-β1)/Smad signaling pathway. Collectively, our findings showed that DAPA could ameliorate cardiac fibrosis in normoglycemic CHF rabbits by inhibiting the TGF-β1/Smad signaling pathway.

## Background

In the past 30 years, despite significant progress in heart failure (HF) treatment, the outcomes often remain unsatisfactory. Due to aging, the overall incidence of HF is increasing in developing countries (Dunlay and Roger, 2014; Roth et al., 2015; Savarese and Lund, 2017; Conrad et al., 2018). The prevalence of HF is about 1–2% of the adult population (Savarese and Lund, 2017; Conrad et al., 2018), with 1-year all-cause mortality rates of 17 and 7% and 1-year hospitalization rates of 44 and 32% for hospitalized and stable/ambulatory HF patients, respectively, (Maggioni et al., 2013). The overall prognosis of HFmrEF (heart failure with mid-range ejection fraction) and HFpEF (heart failure with preserved ejection fraction) is better than that of HFrEF (heart failure with reduced ejection fraction) (Chioncel et al., 2017). In contrast, the transition in ejection fraction is frequently seen over time.

Sodium-glucose transport protein 2 (SGLT2) is primarily expressed in the S1 and S2 segments of the kidney proximal tubule, where 90% of primary urinary glucose is resorbed (Ferrannini and Solini, 2012; Rieg et al., 2014). SGLT2 inhibitors reduce the blood sugar level by inhibiting SGLT2 on the renal tubules and increasing the amount of sugar loss in the urine. In addition, several recent studies have suggested that SGLT2 inhibitors have a cardioprotective effect, such as the EMPA-REG OUTCOME (Empagliflozin Cardiovascular Outcome Event Trial in Type 2 Diabetes Mellitus Patients–Removing Excess Glucose) trial and DECLARE TIMI 58 (Dapagliflozin Effect on Cardiovascular Events–Thrombolysis in Myocardial Infarction 58) trial (Zinman et al., 2015; Wiviott et al., 2019). Furthermore, SGLT2 inhibitors have been found to reduce the risk of worsening HF or death regardless of the presence or absence of diabetes mellitus (McMurray et al., 2019). However, the detailed mechanisms underlying the cardioprotective effects in normoglycemic HF patients remain largely unexplored.

Myocardial fibrosis is an important pathophysiological mechanism of HF, which is characterized by an increase in the extracellular matrix (ECM). The ECM in heart tissue mainly comprises collagen type I and III, and the proportion of the ECM is closely correlated with the extent of impaired left ventricular ejection fraction (LVEF) (Boluyt and Bing, 2000). The cardiac ECM is produced mainly by cardiac fibroblasts (CFs) and is responsible for ECM homeostasis. The human transforming growth factor-beta (TGF-β) superfamily contains at least 33 members, including TGF-β isoforms, activins, bone morphogenetic protein, and growth differentiation factor, which stimulate the synthesis of ECM proteins by activating Smad2 or Smad3 signaling (Huang et al., 2019). As one of the TGF-β isoforms, TGF-β1 has been shown to play an essential role in the pathogenesis of myocardial fibrosis (Brooks and Conrad, 2000; Nakajima et al., 2000; Schultz et al., 2002). Furthermore, overexpression of TGF-β1 in transgenic mice results in both interstitial fibrosis and hypertrophic growth of cardiac myocytes (Rosenkranz et al., 2002).

A previous study has revealed that the SGLT2 inhibitor empagliflozin ameliorates myocardial fibrosis by inhibiting the formation and deposition of collagen *via* the TGF-β1/Smad pathway in the mouse model of type 2 diabetes mellitus (T2DM) (Li et al., 2019). However, little is known about whether an SGLT2 inhibitor can prevent cardiac fibrosis and inhibit the TGF-β1/Smad pathway in a normoglycemic animal model of chronic heart failure (CHF).

In the present study, we assessed the efficacy of the SGLT2 inhibitor dapagliflozin (DAPA) in improving left ventricular dysfunction (LVD) and myocardial structural damage induced by aortic constriction. In addition, we further evaluated its effect on the TGF-β1/Smad pathway.

## Methods

### Ethical Statement

All the animal-related protocols complied with the Guide for the Care and Use of Laboratory Animals (National Research Council, 2011). This study was approved explicitly by the Ethics Committee of Hebei General Hospital (No. 2021100).

### Animals

A total of 24 New Zealand white rabbits (male, 3.0–3.5 kg, 6 months old) were purchased from Tong Hui Breeding Co., Ltd. (License No. SCXK 2016–002). All rabbits were raised individually in standard cages under standard conditions (room temperature; 12-h light/dark cycle) and adapted for 1 week before further analysis. The animals were given free access to water and 200 g of diet per rabbit.

### Grouping

All rabbits were randomly divided into four groups as follows: 1) sham operation group (sham group, *n* = 6), 2) HF group (*n* = 6), 3) perindopril + HF group (Peri group, *n* = 6), and 4) DAPA + HF group (DAPA group, *n* = 6).

### Modeling of CHF

The rabbits were anesthetized with a 3% sodium pentobarbital solution. The chest was opened between intercostal 2 and 3 at the left sternal border area, and the ascending aorta was exposed. The ascending aorta was dissected free for about 4–5 mm at 1.0 cm distal to the aortic root. The aortic circumference was measured, and then it was ligated with silk thread to 60% of its original circumference. HF was considered successfully established once the LVEF (%) was ≤ 40%. The number of rabbits that survived and met the criteria of HF at 12 weeks post aortic coarctation was six in the sham group, five in the HF group, five in the Peri group, and five in the DAPA group. In the sham group, the rabbits only received thoracotomy. In the HF group, rabbits underwent thoracotomy and ascending aortic cerclage. Both the sham and HF groups were given normal saline *via* gavage. In the Peri or DAPA group, the rabbits received thoracotomy and ascending aortic cerclage and then perindopril (0.5 mg/kg/day) or DAPA (1 mg/kg/day) was given by oral gavage for 10 weeks after HF. DAPA, a selective SGLT2 inhibitor, was provided by AstraZeneca Pharmaceuticals LP (America).

### Echocardiographic Evaluation

The Philips EPIQ7 ultrasonic diagnostic system (Philips Ultrasound, Inc.) and S5-1 probe at 1–5 MHz frequency were used to measure the LV function of rabbits after being anesthetized with a 3% sodium pentobarbital solution. Data were collected from at least three cardiac cycles. Left ventricular endsystolic diameter (LVESD), left ventricular end-diastolic diameter (LVEDD), interventricular septal (IVS) thickness, left ventricular posterior wall (LVPW) thickness, fraction shortening (FS), and LVEF were measured before the operation (*t* = 0 weeks), 12 weeks after the operation (*t* = 12 weeks), and 10 weeks of drug treatment (*t* = 22 weeks).

### Measurements of Plasma N-Terminal Pro-Brain Natriuretic Peptide

Plasma NT-proBNP levels were determined before euthanasia using a double-antibody sandwich ABC-ELISA kit (Jingmei Biological Technology Co., Ltd., Jiangsu Province, China).

### Measurements of Plasma Biochemical Indicators, Body Weight, Whole Heart Mass, and LV Mass

After 10 weeks of drug treatment, blood was collected from the ear vein of all rabbits before euthanasia for the analysis of biochemical indicators using a biochemical analyzer (American Beckman Coulter Co., Ltd.). The BW of all rabbits was also measured before euthanasia. The whole heart mass was measured exactly after the heart was removed. After cutting off the right ventricle, atria, and aorta, the LV mass was accurately measured, and the LV mass/BW ratio was calculated.

### Histology and Immunohistochemistry

The rabbits were euthanized at the end of the experiments, and their hearts were dissected. The left ventricular apex was subjected to fixation in 4% paraformaldehyde for 48 h and dehydrated. The fixed tissue was embedded in paraffin and cut into 4-μm sections. Myocardial tissue sections were subjected to Masson’s trichrome staining to assess the severity of myocardial fibrosis. The connective tissue area was determined, which was divided by the sum of the connective tissue area and muscle area to obtain the collagen volume fraction (CVF).

The levels of TGF-β1, collagen I, and collagen III in the tissue were also quantitatively evaluated by the IHC assay. Dewaxed slides were subjected to antigen retrieval in 10 mM sodium citrate (pH 6.0) solution at 100°C for 10 min, followed by incubation in 3% hydrogen peroxide. The slides were incubated with primary antibodies against TGF-β1, collagen I, or collagen III (BIOS Beijing, China) for 1.5 h. Subsequently, the slides were incubated with the corresponding secondary antibody at room temperature for 2.5 h. The slides were then washed with PBS thrice and incubated in 0.02% diaminobenzidine for 2–8 min. After counterstaining with hematoxylin, the slides were briefly washed with resinene and examined under an optical microscope.

### Analysis of Myocardial Fibrosis and the TGF-β1/Smad Pathway

RIPA tissue lysis buffer, PMSF, protease inhibitor, and phosphoprotease inhibitor were added to the rabbit myocardial tissue at the ratio of 100:1:1:1, respectively, and tissue lysis was carried out on ice. The total protein concentration was quantified by a BCA protein determination kit (Solarbio, Beijing, China). Briefly, equal amounts of proteins (30 μg) were subjected to SDS-PAGE and then transferred onto the PVDF membranes. The membranes were blocked with 5% skim milk and incubated with primary antibodies (collagen Ibs-10423R 1: 800, collagen IIIbs-0549R 1: 800, α-SMAbs-0189R 1: 500, CTGFbs-22361R 1: 1,000, TGF-β1+2 + 3 bs-4538R 1: 1,000, Smad2bs-0718R 1:1,000, Smad39531 1:800, Smad7SC-1001152 1:800, MMP2bs-4605R 1:1,500, MMP9bs-4593R1:1,000, TIMP1bs-0415R 1:1,000, and β-actin 66009-1-Ig 1:5,000 Bioss, Beijing, China) overnight at 4°C. The membranes were washed and then incubated with horseradish peroxidase–labeled secondary antibody at room temperature for 1 h. The immunoreactive bands were visualized using ImageJ (version 1.8.0), and β-actin was adopted as a loading control.

### Statistical Analysis

Statistical analysis was performed using SPSS 26.0 statistical software. The results were expressed as mean and standard deviation (SD). Multigroup comparisons were carried out by one-way analysis of variance, followed by the least significant difference test. A *p* < 0.05 was considered statistically significant.

### Results

At 12 weeks after aortic constriction, 21 rabbits survived and met the criteria of HF, including six in the sham group, five in the HF group, five in the Peri group, and five in the DAPA group.

### Effects of DAPA on the Characteristics and Biochemical Indicators of Rabbits

**FIGURE 1:**
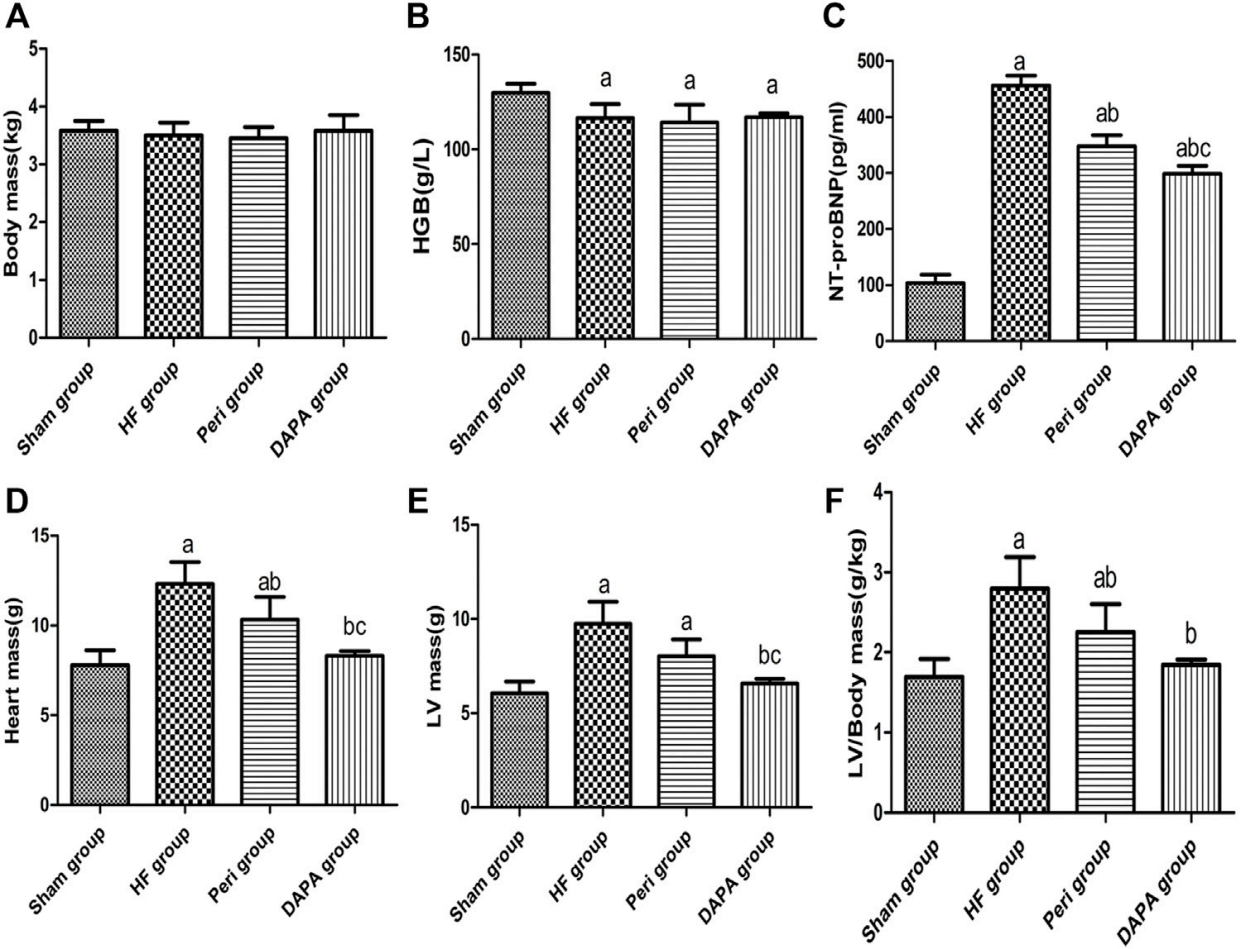
Characteristics and biochemical indicators with statistical differences after 10 weeks of treatment in four groups. ^a^ *p* < 0.05 vs. the sham group; ^b^ *p* < 0.05 vs. the HF group; ^c^ *p* < 0.05 vs. the Peri group.

Table 1 summarizes the biochemical indicators of the four groups. There was no statistical difference in body weight (Figure 1A), random blood glucose, sodium concentration, plasma creatinine, and osmotic pressure. Compared with the sham group, the hemoglobin level in the HF group, Peri group, and DAPA group was decreased (*p* < 0.05), while there was no statistical difference in hemoglobin among the latter three groups (Figure 1B). Meanwhile, the NT-proBNP level was significantly reduced after 10 weeks of DAPA or perindopril treatment compared with that in the HF group (*p* < 0.05). Furthermore, DAPA could more dramatically reduce the NT-proBNP level than perindopril (*p* < 0.05) (Figure 1C). Compared with the sham group, the heart mass, LV mass, and LV mass/BW ratio in the HF group and Peri group were significantly increased (*p* < 0.05). Perindopril could reduce the heart mass and LV mass/BW ratio in rabbits with HF, while there was no significant difference in the LV mass. However, DAPA significantly reduced the heart mass, LV mass, and LV mass/BW ratio, showing a better protective effect than perindopril (Figures 1D–F).

**TABLE 1:** Characteristics and biochemical indicators of rabbits after 10 weeks of treatment in four groups.

	Sham group	HF group	Peri group	DAPA group	F	P
N	6	5	5	5		
BW (kg)	3.58 ± 0.17	3.49 ± 0.23	3.58 ± 0.27	3.45 ± 0.19	0.475	0.704
Glucose (mmol/L)	6.13 ± 0.50	6.08 ± 0.28	6.80 ± 0.59	6.38 ± 0.42	2.573	0.088
Na+(mmol/L)	143.17 ± 1.47	143.00 ± 1.58	141.40 ± 1.82	141.60 ± 1.14	1.940	0.161
HGB (g/L)	129.83 ± 4.71	116.40 ± 7.44^a^	114.20 ± 9.26^a^	116.80 ± 2.17^a^	7.055	0.003
NT-proBNP (pg/ml)	104.40 ± 9.72	449.70 ± 21.26^a^	352.20 ± 15.39^ab^	298.55 ± 14.31^abc^	495.557	0.001
Creatinine (μmol/L)	99.47 ± 14.48	100.72 ± 15.04	109.32 ± 6.96	106.38 ± 12.71	0.701	0.564
Osmotic pressure	305.81 ± 3.58	307.88 ± 3.38	303.16 ± 2.86	304.09 ± 5.17	1.481	0.255
Heart mass (g)	7.78 ± 0.83	12.31 ± 1.21^a^	10.32 ± 1.27^ab^	8.31 ± 0.26^bc^	22.953	0.001
LV mass (g)	6.05 ± 0.62	9.74 ± 1.17^a^	8.02 ± 0.89^a^	6.56 ± 0.27^bc^	22.683	0.001
LV mass/BW (g/kg)	1.69 ± 0.22	2.79 ± 0.39^a^	2.25 ± 0.35^ab^	1.84 ± 0.07^b^	15.835	0.001

Values were expressed as the mean ± SD. Statistical analyses were conducted by one-way ANOVA, followed by Tukey’s or Games–Howell post hoc test.

a *p* < 0.05 vs. the sham group.

b *p*< 0.05 vs. the HF group.

c *p* < 0.05 vs. the Peri group.

### Effect of DAPA on LV Functions and Remodeling

**FIGURE 2:**
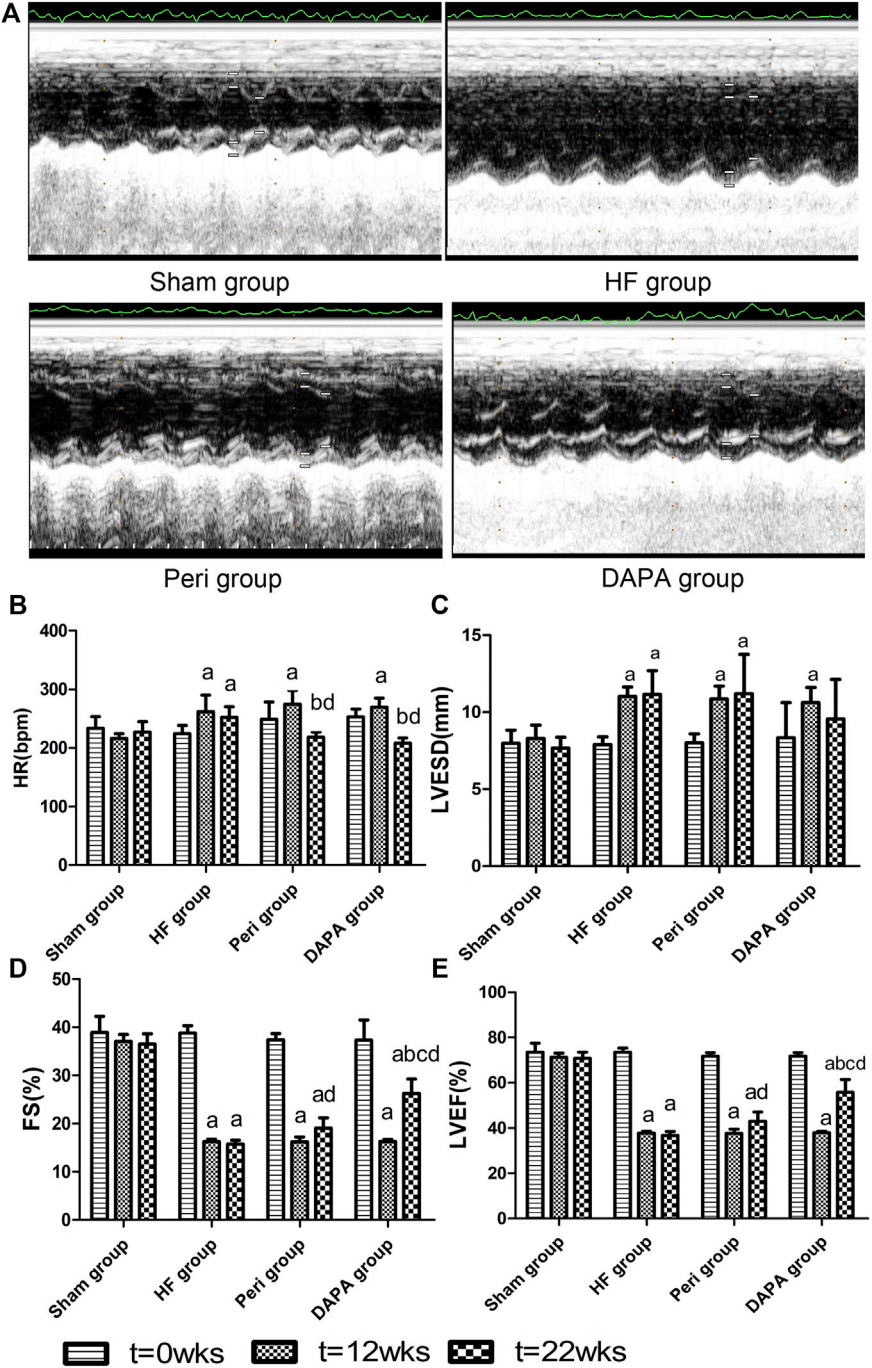
Echocardiographic representative images after drug treatment by M-mode and **(A)** comparison of HR and **(B)** echocardiographic data of the left ventricle **(C–E)**. ^a^ *p* < 0.05 vs. the sham group; ^b^ *p* < 0.05 vs. the HF group; ^c^ *p* < 0.05 vs. the Peri group; ^d^ *p* < 0.05 vs. the counterpart data of the 12th week.

Echocardiography was performed at the time points of 0 weeks, 12th week, and 22nd week (Figure 2, Table 2). There was no statistical difference in the heart rate and all of the echocardiography indexes between the four groups at 0 weeks. After 12 weeks of aortic coarctation, the heart rate and LVESD in the HF group, Peri group, and DAPA group were significantly increased compared with those in the sham group (*p* < 0.05), and FS and LVEF in those groups were significantly lower than those in the sham group (*p* < 0.05). At the 22nd week, the heart rate of the HF group was higher than that in the sham group (*p* < 0.05), and rabbits treated with perindopril or DAPA had a lower heart rate than that in the HF group (*p* < 0.05). Moreover, the heart rate in the Peri group or DAPA group was also significantly decreased from that before the treatment. Nevertheless, there was no remarkable discrepancy between the Peri and DAPA groups (Figure 2B). Figure 2A shows the echocardiogram representation of experimental animals in each group after drug treatment. Perindopril treatment could improve FS and LVEF compared with its baseline (*p* < 0.05), while there was no statistical difference compared with those in the HF group (*p* > 0.05). However, LVESD in the DAPA group was similar to that in the sham group. Meanwhile, FS and LVEF were significantly higher than those in the HF group at the end of the study, which were also higher than their baseline before the treatment (*p* < 0.05). Compared with the Peri group, DAPA could further increase cardiac ejection fraction (Figures 2C–E).

**TABLE 2:** Echocardiographic investigation of left ventricle structural and functional data in rabbits.

	*t* = 0 wks					
	Sham group (*n* = 6)	HF group (*n* = 5)	Peri group (*n* = 5)	DAPA group (*n* = 5)	*F*	*P*
HR (bpm)	233.5 ± 19.9	224.6 ± 13.8	248.8 ± 29.7	253.2 ± 13.2	2.191	0.126
IVS (mm)	2.83 ± 0.09	3.02 ± 0.29	2.81 ± 0.13	2.89 ± 0.14	1.337	0.284
LVEDD (mm)	13.05 ± 1.27	12.88 ± 0.85	12.80 ± 1.05	13.19 ± 3.12	0.049	0.985
LVPW (mm)	2.77 ± 0.19	3.02 ± 0.17	2.79 ± 0.10	2.71 ± 0.28	2.369	0.107
LVESD (mm)	7.97 ± 0.85	7.88 ± 0.51	8.01 ± 0.58	8.33 ± 2.29	0.125	0.944
FS (%)	38.90 ± 3.35	38.82 ± 1.54	37.38 ± 1.31	37.36 ± 4.15	0.468	0.708
EF (%)	73.43 ± 4.12	73.54 ± 1.85	71.78 ± 1.51	71.48 ± 1.52	0.470	0.707
	***t* = 12 wks**					
	**Sham group (*n* = 6)**	**HF group (*n* = 5)**	**Peri group (*n* = 5)**	**DAPA group (*n* = 5)**	***F***	***P***
HR (bpm)	216.2 ± 8.2	262.2 ± 28.1^a^	274.2 ± 24.1^a^	269.6 ± 15.3^a^	10.195	0.001
IVS (mm)	2.81 ± 0.11	2.97 ± 0.17	3.02 ± 0.27	3.09 ± 0.21	2.075	0.142
LVEDD (mm)	13.18 ± 1.33	13.16 ± 0.78	12.96 ± 1.01	12.70 ± 1.20	0.212	0.887
LVPW (mm)	2.79 ± 0.09	2.91 ± 0.24	2.79 ± 0.16	2.84 ± 0.06	0.734	0.546
LVESD (mm)	8.29 ± 0.87	11.02 ± 0.61^a^	10.86 ± 0.84^a^	10.62 ± 0.99^a^	13.281	0.001
FS (%)	37.08 ± 1.38	16.22 ± 0.52^a^	16.20 ± 0.99^a^	16.32 ± 0.37^a^	696.253	0.001
EF (%)	71.32 ± 1.77	37.70 ± 0.89^a^	37.62 ± 1.94^a^	37.98 ± 0.67^a^	767.830	0.001
	***t* = 22 wks**					
	**Sham group (*n* = 6)**	**HF group (*n* = 5)**	**Peri group (*n* = 5)**	**DAPA group (*n* = 5)**	***F***	***P***
HR (bpm)	227.1 ± 18.0	252.2 ± 18.3^a^	218.2 ± 9.0^bd^	208.2 ± 9.0^bd^	8.209	0.001
IVS (mm)	2.92 ± 0.17	2.92 ± 0.17	2.99 ± 0.56	2.79 ± 0.30	0.321	0.810
LVEDD (mm)	12.07 ± 1.16	13.24 ± 1.81	13.84 ± 3.12	12.86 ± 2.84	0.566	0.645
LVPW (mm)	2.79 ± 0.09	2.79 ± 0.10	2.71 ± 0.21	2.71 ± 0.23	0.397	0.757
LVESD (mm)	7.65 ± 0.71	11.16 ± 1.54^a^	11.21 ± 2.54^a^	9.55 ± 2.57	4.156	0.022
FS (%)	36.51 ± 2.15	15.72 ± 0.86^a^	19.06 ± 2.11^ad^	26.28 ± 2.99^abcd^	100.528	0.001
EF (%)	70.82 ± 2.65	36.70 ± 1.72^a^	42.98 ± 4.04^ad^	55.82 ± 5.59^abcd^	90.073	0.001

Data were expressed as mean ± SD. Statistical analyses were conducted by one-way ANOVA, followed by the Tukey’s or Games–Howell post hoc test.

a *p* < 0.05 vs. the sham group.

b *p*< 0.05 vs. the HF group.

c *p* < 0.05 vs. the Peri group.

d *p* < 0.05 vs. the counterpart data of the 12th week.

### Effect of DAPA on Myocardial Fibrosis

**FIGURE 3:**
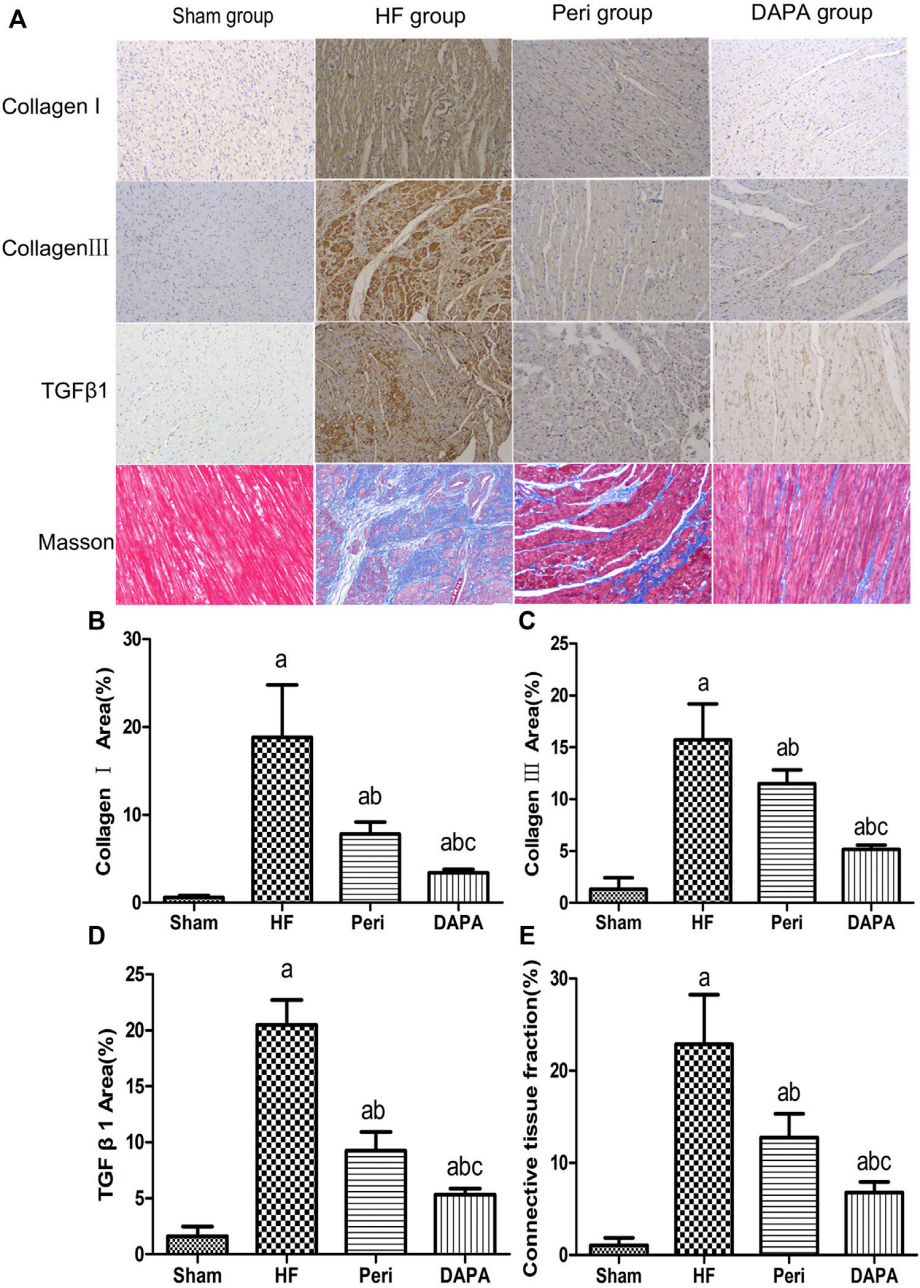
DAPA suppresses myocardial fibrosis in the CHF rabbit model. IHC staining of collagen I, collagen III, and TGF-β1 and Masson’s trichrome staining of the myocardium. **(A)** Percentages of positive areas of collagen I **(B)**, collagen III **(C)**, TGF-β1 , and **(D)** connective tissue fraction. **(E)** Data were expressed as the mean ± SD. ^a^ *p* < 0.05 vs. the sham group; ^b^ *p* < 0.05 vs. the HF group; ^c^ *p* < 0.05 vs. the Peri group.

IHC staining of collagen Ⅰ and collagen Ⅲ showed that the number of positive cells of collagen Ⅰ and collagen Ⅲ in the HF group was significantly increased compared with that in the sham group. Compared with the HF group, perindopril and DAPA markedly reduced the expressions of collagen Ⅰ and collagen Ⅲ (*p* < 0.05). Moreover, DAPA could further reduce the expressions of collagen I and III compared with perindopril (*p* < 0.05). Similarly, the IHC assay revealed significant differences in the expression of TGF-β1 among the four groups (all *p* < 0.05). DAPA could further inhibit the expression of TGF-β1 compared with that of perindopril (*p* < 0.05) (Figures 3A–D).

Figure 3A shows that cardiac interstitial fibrosis could be assessed by Masson’s trichrome staining, and the severity of myocardial fibrosis could be quantified as a connective tissue fraction. The degree of LV fibrosis in the HF group, Peri group, and DAPA group was significantly higher than that in the sham group. Compared with the HF group, LV fibrosis was significantly suppressed by perindopril and DAPA. Moreover, DAPA was superior to perindopril in inhibiting myocardial fibrosis (*p* < 0.05) (Figure 3E).

### Effect of DAPA on the Expressions of Collagen I, Collagen III, α-SMA, and CTGF

**FIGURE 4:**
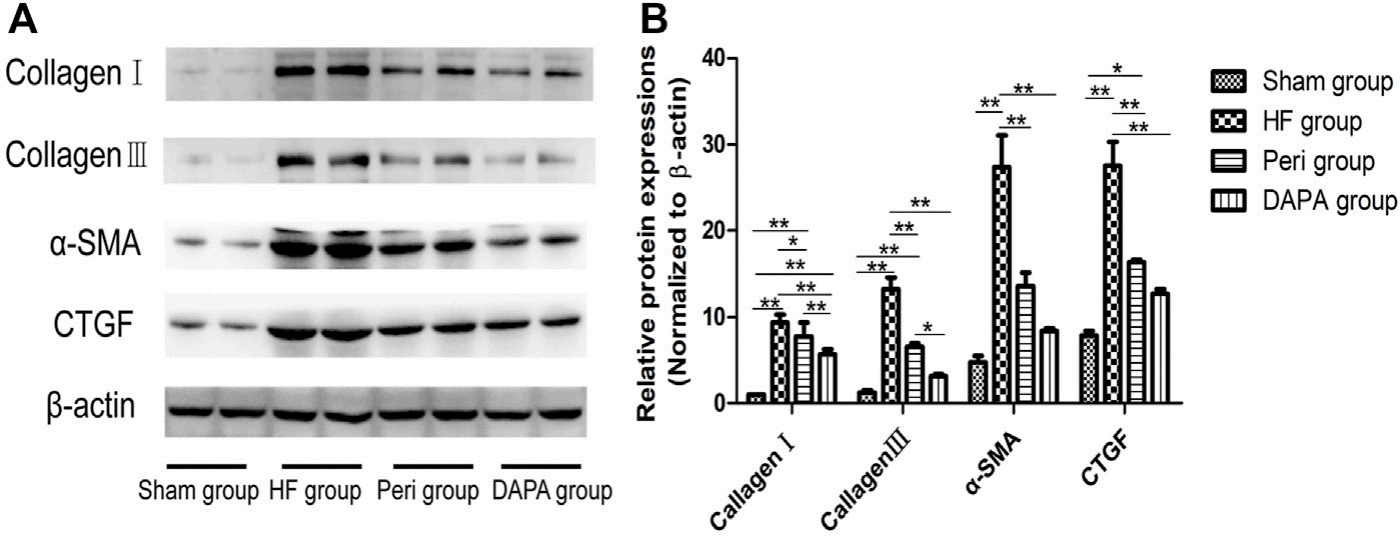
Effect of DAPA on myocardial fibrosis. **(A)** Western blotting analysis for the expressions of collagen I, collagen III, α-SMA, and CTGF. **(B)** Relative expression at the protein level was determined, and β-actin was adopted as the loading control. Data were expressed as the mean ± SD. *p < 0.05; **p < 0.01.

The main features of myocardial fibrosis include the deposition of collagen Ⅰ and collagen Ⅲ in the interstitium of the myocardium. The transformation from fibroblasts into an active fibroblast phenotype or myofibroblasts plays a critical role in the pathogenesis of cardiac fibrosis (Desmoulière et al., 1993). α-smooth muscle actin (α-SMA) is a noticeable indicator for the transition between fibroblasts and myofibroblasts. Meanwhile, myofibroblasts are more likely to be the primary source of connective tissue growth factor (CTGF) (Watsky et al., 2010), which, together with BNP and α-SMA, are regarded as hypertrophy- and HF-related genes (Koitabashi et al., 2007; Daniels et al., 2009). Our study revealed that (Figures 4A,B) the levels of collagen I and collagen III, α-SMA, and CTGF in the HF group were higher than those in the sham group (*p* < 0.01). Both perindopril and DAPA could significantly suppress the upregulation of these genes (*p* < 0.05). Furthermore, the expressions of collagen I and collagen III in the DAPA group were lower than those of the Peri group (*p* < 0.01, or *p* < 0.05). The levels of α-SMA and CTGF in the DAPA group were also lower than those of the Peri group, while there were no statistical differences between the two groups (*p* > 0.05), indicating that DAPA could suppress myocardial fibrosis and play a superior role compared with perindopril.

### Effect of DAPA on the TGF-β1/Smad Pathway in the Heart of Rabbits

**FIGURE 5:**
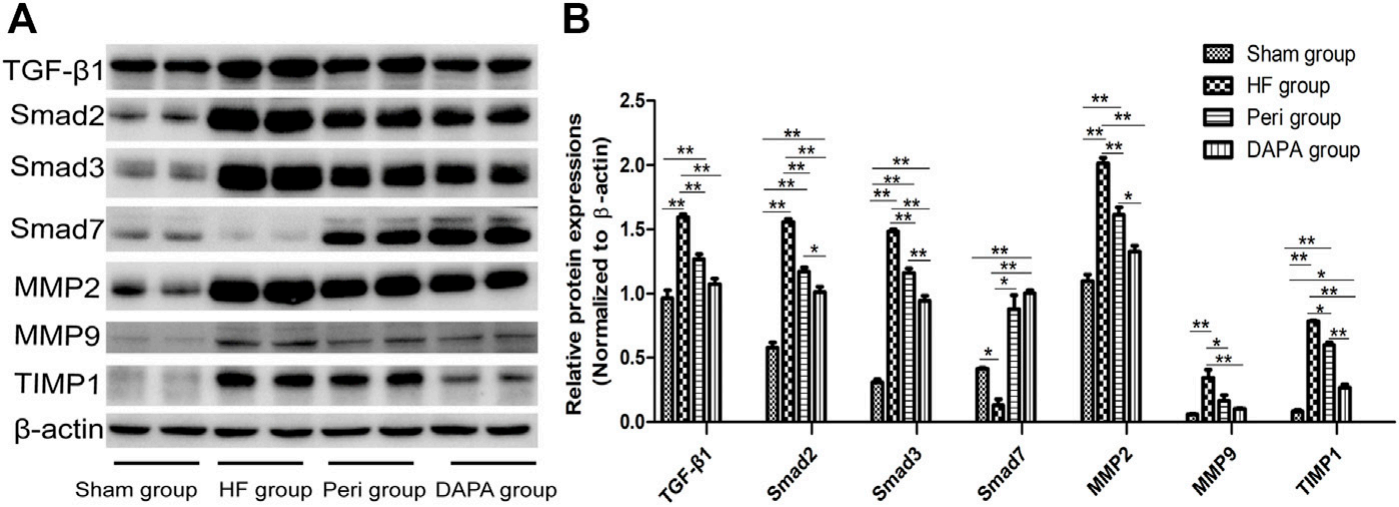
Effect of DAPA on the TGF-β1/Smad pathway *in vivo*. **(A)** Western blotting analysis for the expressions of the TGF-β1/Smad pathway. **(B)** Relative expression at the protein level was determined, and β-actin was adopted as a loading control. Data were expressed as the mean ± SD. *p < 0.05; **p < 0.01.

In the present study, TGF-β1/Smad signaling was investigated in the four groups using Western blotting analysis (Figures 5A,B). TGF-β1/Smad signaling was more significantly activated in the heart tissue of HF rabbits than in the sham group, evidenced by remarkably upregulated expressions of TGF-β1, Smad2, and Smad3 (*p* < 0.01). Both perindopril and DAPA could inhibit the upregulation of TGF-β1, Smad2, and Smad3 (*p* < 0.01), and DAPA was superior to perindopril in terms of inhibiting the TGF-β1/Smad signaling (*p* < 0.01, or *p* < 0.05). As a negative inhibitor of TGF-β1/Smad signaling, Smad7 was downregulated in the HF group (*p* < 0.05), while it was significantly upregulated in the Peri group (*p* < 0.05) and DAPA group (*p* < 0.01). DAPA treatment could more significantly increase the expression of Smad7 than that of the Peri group, although there was no statistical difference (*p* > 0.05).

The myocardial expressions and activities of matrix metalloproteinases (MMPs) are elevated upon pressure overload (Sakata et al., 2004). The levels of MMP2 and MMP9 in the HF group were significantly increased compared with those in the sham group (*p* < 0.01). TIMP1 was also upregulated to maintain the balance (*p* < 0.01). DAPA could reduce the expressions of MMP2, MMP9, and TIMP1 (*p* < 0.05), showing a superior protective effect compared with perindopril.

## Discussion

As a highly selective inhibitor of SGLT2, dapagliflozin can ameliorate T2DM by enhancing renal glucose excretion or glycosuria (Garcia-Ropero et al., 2018). However, quite unexpectedly, the SGLT2 inhibitor can reduce major adverse cardiovascular events, including cardiovascular death, HF-related admission, and all-cause mortality, in recent trials (Zinman et al., 2015; Neal et al., 2017; McMurray et al., 2019; Wiviott et al., 2019). In those individuals with HF and impaired ejection fraction, dapagliflozin can more significantly reduce the risk of worsening HF or death due to CVD than the placebo, regardless of the presence or absence of diabetes (McMurray et al., 2020). Therefore, dapagliflozin or empagliflozin has been recommended for patients with HFrEF regardless of diabetes status to reduce the risk of HF hospitalization and death (McDonagh et al., 20212021). Many studies have explored the potential mechanisms of the cardiac effects of these agents. Xuan Li et al. (Li et al., 2021) have reported that SGLT2i empagliflozin can increase exercise endurance and survival rate, improve LV systolic and diastolic function, and attenuate adverse LV remodeling and cardiac fibrosis in mice with transverse aortic constriction–induced HF. The mechanism may be related to reducing glycolysis, rebalancing coupling between glycolysis and oxidative phosphorylation by directly binding to cardiac glucose transporters, and regulating the adenosine monophosphate–activated protein kinase mammalian target of the rapamycin complex 1 pathway. Another SGLT2i dapagliflozin can also improve LVEF in rats with HF induced by volume overload by suppressing cardiac fibrosis and endoplasmic reticulum stress and improving hemodynamics (Lin et al., 2021). Moreover, studies (Cappetta et al., 2020; Withaar et al., 2021) have shown that dapagliflozin can improve cardiac structure and function in non-diabetic mouse models of HF with preserved ejection fraction. The protective effect against HFpEF may be exerted by reducing diastolic Ca^2+^ and Na^+^ overload, increasing Ca^2+^ transient amplitude in ventricular cardiomyocytes, reversing endothelial activation and endothelial nitric oxide synthase deficit, inhibiting cardiac inflammation or profibrotic signaling, and attenuating cardiometabolic dysregulation. Meanwhile, the effect of reducing sympathetic nervous system overdrive, promoting weight loss, and decreasing the levels of uric acid and triglyceride may be responsible for their cardioprotective effects (Zelniker and Braunwald, 2020). However, despite numerous experimental and preclinical studies that have been conducted, the details of the molecular pathways mediating the DAPA effect on normoglycemic HF patients remain lacking.

In the present investigation, we assessed the effect of the SGLT2 inhibitor DAPA on cardiac function and remodeling in normoglycemic rabbits with CHF. Moreover, we also explored the possible underlying mechanisms.

We confirmed that DAPA could effectively improve heart function and attenuate cardiac remodeling in normoglycemic rabbits with CHF, and the underlying mechanism might not be related to the following factors, namely, glucose-lowering, BW, uric acid or creatinine levels, and osmotic diuresis and natriuresis. Moreover, DAPA ameliorated cardiac fibrosis and reduced ECM proteins, including collagen I, collagen III, and α-SMA, by suppressing TGF-β1/Smad signaling. Our findings further supported the fact that DAPA could be prescribed for normoglycemic CHF patients to relieve their symptoms.

Cardiac fibrosis remains a critical hallmark of HF, which is featured by the upregulation of collagens and other ECM components in the interstitium and perivascular regions of the myocardium (Berk et al., 2007). The fibrillar collagens, type I and III, are the most abundant collagens in the heart, accounting together for over 90% of the total collagen. The severity of interstitial fibrosis is tightly associated with the extent of LV hypertrophy and impaired ejection fraction in the pressure-overloaded heart (Boluyt and Bing, 2000). A previous study has indicated that CTGF can induce the proliferation of CFs and enhance ECM production (Wang et al., 2008), and CTGF serves as a noticeable marker once fibroblasts are activated during myocardial fibrosis (Bonniaud et al., 2004; Hayata et al., 2008). In addition, another characteristic of activated fibroblasts is the expression of α-SMA (Hinz and Lagares, 2020). Accumulating evidence has shown that TGF-β1 plays an essential role in the ECM metabolism in a variety of organ systems (Massagué, 1990) and in the mouse models of HF (Nakajima et al., 2000; Schultz et al., 2002). TGF-β1 is also significantly upregulated in the pressure-overloaded human heart (Villar et al., 2009) and strongly contributes to fibrotic disorders in heart disease (Theron et al., 2017). It is well known that Smad functions as a downstream regulator of TGF-β1 signaling (Pardali and Ten Dijke, 2012). TGF-β1/Smad signaling serves as a classical cell signaling pathway in the disease progression. Smad proteins can be categorized into three groups: receptor-activated Smads (Smad1, Smad2, Smad3, Smad5, and Smad8), co-mediator Smads (Smad4 and Smad10), and inhibitory Smads (Smad6 and Smad7) (Pardali and Ten Dijke, 2012). Smad2 and Smad3 are phosphorylated by activated TGF-β1, forming a Smad complex by combining with Smad4. Such a complex is transported into the nucleus, in which it, together with other transcription factors, co-activators, and co-repressors, modulates the expressions of target genes, including collagen I, collagen III, α-SMA, and MMPs (Zhu et al., 2013). However, inhibitory Smads, such as Smad7, suppress R-Smad activation either by competing with R-Smads for type I receptor interaction and/or by recruiting specific ubiquitin ligases or phosphatases to the activated receptor complex.

Previous *in vitro* and *in vivo* studies have shown that angiotensin Ⅱ can enhance the expression of TGF-β1 in myofibroblasts and CFs (Lee et al., 1995; Campbell and Katwa, 1997). Angiotensin Ⅱ antagonists suppress the expression of TGF-β1 in cardiac and vascular tissues in rats (Kim et al., 1995). Likewise, angiotensin Ⅱ receptor blockers can inhibit TGF-β1 and restrain myocardial fibrosis in rat models of hypertension. Further investigation has suggested that hypertrophy cannot be induced by angiotensin Ⅱ in TGF-β1–deficient mice (Schultz et al., 2002). Moreover, clinical studies have shown that the serum level of TGF-β1 is decreased by angiotensin-converting enzyme (ACE) inhibitors and angiotensin Ⅱ receptor antagonists (Laviades et al., 2000; Agarwal et al., 2002). Collectively, ACE inhibitors and angiotensin II receptor blockers are likely to exert their ameliorative effects *via* antagonism of TGF-β1 and its downstream proteins. As one of the representative drugs of ACEI, perindopril has been widely used to treat HF and inhibit myocardial fibrosis. In the present study, we verified that perindopril could suppress myocardial fibrosis and myocardial remodeling by inhibiting the TGF-β1-Smad signaling pathway. Moreover, we proved that DAPA also exerted an inhibitory effect on the TGF-β1-Smad signaling pathway, showing a better cardioprotective effect than that of perindopril.

Proteolytic enzymes, such as MMPs, play a central role in cardiac remodeling. Both MMPs and their endogenous inhibitors, TIMPs, are involved in the ECM turnover mediated by CFs. Accumulating evidence suggests that TGF-β1 enhances the activities of MMPs within the myocardium. Moreover, previous studies have indicated that TGF-β1 upregulates the expressions of MMP2 and MMP9 (Overall et al., 1991; Shimizu et al., 1998; Briest et al., 2004). TIMPs are also involved in the regulation of MMP activity. The equilibrium between TIMPs and MMPs plays an essential role in myocardial fibrosis. Therefore, it is an important therapeutic target to recover the equilibrium of TIMPs and MMPs for HF patients. Encouragingly, DAPA has such an effect on the myocardium of CHF patients without T2DM.

In conclusion, our study demonstrated that the expressions of collagen I, collagen -III, α-SMA, and CTGF were remarkably upregulated once the HF model was established, while DAPA could markedly downregulate these proteins, showing a better cardioprotective effect than that of perindopril.

## Conclusion

In summary, we showed that 10 weeks of DAPA treatment increased cardiac ejection fraction and attenuated myocardial fibrosis in normoglycemic CHF rabbits, and the cardioprotective effect of DAPA was superior to perindopril. Furthermore, our results suggested that DAPA suppressed collagen formation and deposition *via* the classical TGF-β1/Smad pathway to attenuate myocardial fibrosis. Moreover, its downstream molecules, such as MMPs, were also involved in such a process. Therefore, our findings collectively provided evidence that DAPA exerted a cardioprotective effect against HF, at least partially, by mediating the TGF- β1 /Smads signaling pathway. However, more clinical data are needed to verify the safety and efficacy of dapagliflozin in normoglycemic patients with CHF.

## Data Availability

The original contributions presented in the study are included in the article/Supplementary Material, further inquiries can be directed to the corresponding author.
