# Supplementary material for: Dapagliflozin Attenuates Myocardial Fibrosis by Inhibiting the TGF-β1/Smad Signaling Pathway in a Normoglycemic Rabbit Model of Chronic Heart Failure
Source: Front Pharmacol. 2022 May 13;13:873108. doi: 10.3389/fphar.2022.873108 (PMC9136228; doi:10.3389/fphar.2022.873108)
Supplement: Supplementary file 1 [file DataSheet1.PDF]

# Dapagliflozin Attenuate Myocardial Fibrosis by Inhibiting TGF-β1/ Smad Signaling Pathway in Non diabetic Rabbit Model of Chronic Heart Failure

Xuefeng Chen[1,2], Qian Yang[2], Wenlou Bai[2], Wenjing Yao[2], Litian Liu[2], Yuanyuan Xing[2], Cunliang Meng[2], Peng Qi[2], Yi Dang [2], Xiaoyong Qi[1,2*]

[1] Department of Internal Medicine, Hebei Medical University, Shijiazhuang 050000, Hebei Province, People's Republic of China

[2] Department of Cardiology Center, Hebei General Hospital, Shijiazhuang 050000, Hebei Province, People's Republic of China

*** Corresponding author:**

**Xiaoyong Qi**

**Email:** xiaoyongqi2021@126.com

**Tel:** 0-13603211006

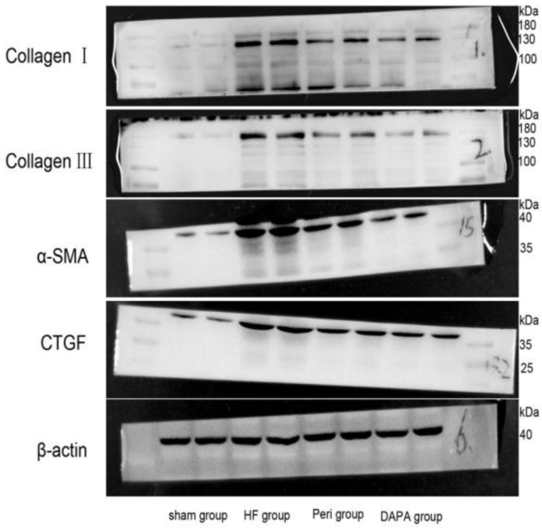


Original western blots of 3A

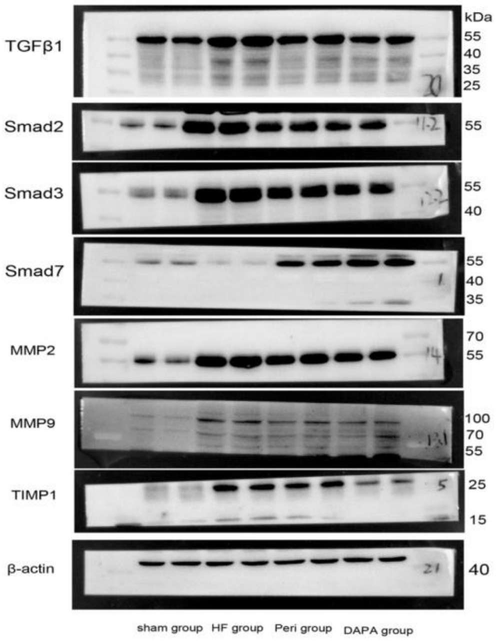


Original western blots of Figure 4A
